# Sounds Scary? Lack of Habituation following the Presentation of Novel Sounds

**DOI:** 10.1371/journal.pone.0014549

**Published:** 2011-01-18

**Authors:** Tine A. Biedenweg, Michael H. Parsons, Patricia A. Fleming, Daniel T. Blumstein

**Affiliations:** 1 Department of Environmental and Aquatic Sciences, Curtin University of Technology, Perth, Australia; 2 School of Veterinary and Biomedical Sciences, Murdoch University, Murdoch, Australia; 3 Department of Biology, State University of New York - Empire State College, Hartsdale, New York, United States of America; 4 Department of Ecology and Evolutionary Biology, University of California Los Angeles, Los Angeles, California, United States of America; University of Pretoria, South Africa

## Abstract

**Background:**

Animals typically show less habituation to biologically meaningful sounds than to novel signals. We might therefore expect that acoustic deterrents should be based on natural sounds.

**Methodology:**

We investigated responses by western grey kangaroos (*Macropus fulignosus*) towards playback of natural sounds (alarm foot stomps and Australian raven (*Corvus coronoides*) calls) and artificial sounds (faux snake hiss and bull whip crack). We then increased rate of presentation to examine whether animals would habituate. Finally, we varied frequency of playback to investigate optimal rates of delivery.

**Principal Findings:**

Nine behaviors clustered into five Principal Components. PC factors 1 and 2 (animals *alert* or *looking*, or *hopping* and moving *out of area*) accounted for 36% of variance. PC factor 3 (eating cessation, taking flight, movement out of area) accounted for 13% of variance. Factors 4 and 5 (relaxing, grooming and walking; 12 and 11% of variation, respectively) discontinued upon playback. The whip crack was most evocative; *eating* was reduced from 75% of time spent prior to playback to 6% following playback (post alarm stomp: 32%, raven call: 49%, hiss: 75%). Additionally, 24% of individuals took *flight* and moved *out of area* (50 m radius) in response to the whip crack (foot stomp: 0%, raven call: 8% and 4%, hiss: 6%). Increasing rate of presentation (12x/min ×2 min) caused 71% of animals to *move out of the area*.

**Conclusions/Significance:**

The bull whip crack, an artificial sound, was as effective as the alarm stomp at eliciting aversive behaviors. Kangaroos did not fully habituate despite hearing the signal up to 20x/min. Highest rates of playback did not elicit the greatest responses, suggesting that ‘more is not always better’. Ultimately, by utilizing both artificial and biological sounds, predictability may be masked or offset, so that habituation is delayed and more effective deterrents may be produced.

## Introduction

Populations of large kangaroos (*Macropus* spp.) have flourished following the European settlement of Australia. This upsurge is primarily due to the elimination of primary predators such as the dingo [1] and by the provision of artificial watering points for livestock [2]. Like medium to large sized herbivores on other continents (e.g., elk, *Cervus canadensis* and mule deer, *Odocoileus hemionus* [3]), kangaroos have been implicated for conflicts with farming, forestry and land restoration practices [4]. Currently, there are no non-invasive means to assist the management of kangaroos where they encroach on human habitats [5]. Thus, novel, humane approaches are required to mitigate the effects of herbivory and animal-traffic collisions. If kangaroo behavior can be significantly altered, such as by generating fearful responses to danger cues [6], then impacts may be more naturally managed [7].

When exposed to a fearful stimulus, kangaroos may increase vigilance, decrease foraging and even shift habitat utilization by retreating from high risk areas [7]–[12]. Generally speaking, optical and auditory cues warn of imminent threat, whereas chemosensory signals (kairomones) indicate either past [13] or present [14] predators. The mechanisms by which kangaroos detect danger however, may be species- and/or context- specific [15].

Both olfactory and auditory abilities are well developed in Macropodids due to their crepuscular and/or nocturnal nature [16]. Consequently, semi-wild western grey kangaroos (*Macropus fuliginosus*) respond fearfully following exposure to recent voids of dingo urine (*Canis lupus dingo*) [9]–[10] while eastern grey kangaroos (*M. giganteus*), and the smaller congeneric tammar wallaby (*M. eugenii*), respond to foot stomps – a natural alarm signal [17]–[19]. A better understanding of kangaroo sensitivity to different signals may therefore aid the development of deterrents that utilize a range of modalities.

We have investigated the use of acoustic signals to elicit anti-predator behavior in kangaroos. Firstly, we asked whether artificial or natural signals elicit a greater anti-predator response and may therefore be more appropriate as deterrent signals. Among macropods, natural anti-predator sounds are more effective at generating a fearful response than artificial sounds [17], [20]. In response to playback of foot stomps, conspecifics are more vigilant, and either decrease foraging time or take flight [17]–[19]. However these responses may be species specific: Tammar wallabies increased vigilance and decreased foraging following playback of foot stomps [18]. Eastern grey kangaroos reduce foraging by 74% and increase flight by 26% [17] while captive red-necked pademelons (*Thylogale thetis*) and red-necked wallabies (*M. rufogriseus banksianus*) increased vigilance but did not demonstrate flight [19]. The lack of flight in Ramp's study [19] may have reflected the consequences of research on captive animals in a confined space.

By contrast with natural acoustic signals, artificial sounds such as ultrasonic and infrasonic deterrents have not been particularly effective deterrents for a variety of species in the United States [3] or Australia [17], [20]. Two ultrasonic devices were manufactured to prevent kangaroo vehicle collision (Shu-roo Mk II, Shu Roo Australia Pty Ltd) and to protect agricultural areas (ROO-guard Mk I and II, Shu Roo Australia Pty Ltd). These products have been largely ineffective, presumably because they generate signals outside kangaroo's optimum hearing range [17], [20] and perhaps because of the continuous nature of the signal playback (promoting habituation). Continuous noises may be more easily habituated to, while sounds produced at random intervals, as well as loud, sudden noises, may produce more effective deterrents.

Loud, sudden noises generate startle and flight responses in a wide range of animal species (rats, *Rattus norvegicus* [21]; porpoises, *Phocoena phocoena* [22]; Canada geese, *Branta canadensis* [23]; pigs, *Sus scrofa*, [24]; and rhesus monkeys, *Macaca mulatta* [25]). Sometimes these effects persist without habituation. For instance, coyotes fled target areas following exposure to a startling sound for over 30 days [26], while porpoises show aversive responses to artificial sounds with no habituation over a month [27]. Even when habituation has been evident, minor changes to the signal can again initiate antipredator responses. For example, pigs habituate to an 84 dB SPL animal transporter sound, but minor modifications to the acoustic signal restores the original fearful response [24]. Habituation is therefore, not always permanent.

Secondly, we asked whether kangaroos are more, or less, likely to become habituated to artificial or natural signals. Habituation occurs when short-term memory suppresses the natural response to a recent stimulus [28] and therefore could counteract the long-term benefits of any signal. Previous studies have shown that sounds that provoke the strongest response are expected to take longer to habituate to compared with sounds that initiate a weaker response [21], [29]. Staddon [30] suggests a frightened animal will habituate slowly to any novel sound. However, animals may be much less likely to become habituated to a natural signal that normally is produced in association with the presence of predators as compared with an artificial signal that has no such biological meaning [24], [29]. We therefore predicted that kangaroos are less likely to habituate to kangaroo foot stomps, a natural alarm sound, than to novel sounds.

Thirdly, we asked whether the rate of exposure to a signal affects habituation to the signal. Staddon [30] noted that habituation will increase with the temporal distribution of the stimulus. Similarly, Bomford and O'Brien [31] and Davis [28] found the rate of habituation may be influenced by changing the rate of stimulus, as familiarity (how often the animal hears the sound) often leads to habituation. We therefore predicted greater habituation in experiments where the rate of signal playback was increased.

Together, the results of these questions may lead to the design of more effective acoustic deterrents for kangaroos.

## Results

Kangaroos engaged in a considerable amount of relaxed and feeding behavior in the baseline period prior to experimental treatments. They spent an average of 75% of their time *eating*, 16% of their time *looking* around, 5% of their time *relaxing*, 3% of their time *walking*, and 2% of their time *grooming* (tables 1, 2, 3). The nine behaviors recorded were grouped into five Principal Components (PC) factors (table 4). During the first minute (prior to playback), the only significant difference between behaviors across the experiments was greater relaxing behavior in minute 1 (PC4; relaxed) for experiment 2. This result does not reflect any biological meaningful significance; rather it represents variability in the baseline data.

**Table 1:** Time budget for experiment 1.

Signal	Minute 1 (pre-signal)		Minute 2 (post-signal)		Minute 3 (post-signal)	
Raven call	70.1%	eating	47%	eating	50.6%	eating
	17.6%	looking	43.8%	looking	28.3%	looking
	4.2%	relaxing	5.0%	alert	8.0%	out of area
	3.4%	grooming	2.0%	grooming	7.9%	walking
	2.7%	walking	1.1%	walking	3.4%	alert
	2.0%	alert	0.8%	hopping	1.2%	hopping
	---		0.3%	out of area	0.6%	grooming
Hiss	89%	eating	65.3%	eating	84.4%	eating
	4.8%	looking	25.1%	looking	6.3%	out of area
	4.2%	relaxing	3.8%	out of area	3.3%	walking
	1.2%	grooming	1.8%	walking	1.9%	relaxing
	0.8%	walking	1.3%	hopping	1.6%	looking
			1.0%	grooming	1.3%	grooming
			0.9%	alert	0.9%	alert
			0.2%	flight	0.2%	hopping
Foot stomp	56.9%	eating	45.5%	looking	44.4%	looking
	30.4%	looking	26.5%	alert	41.6%	eating
	5.8%	walking	21.6%	eating	6.9%	alert
	5.1%	grooming	2.6%	walking	6.5%	walking
	1.4%	alert	1.9%	grooming	0.3%	hopping
	0.4%	hopping	0.6%	hopping	0.2%	grooming
	---		0.4%	flight	---	
Whip Crack	71%	eating	41.7%	alert	33.8%	alert
	13.5%	relaxing	19%	out of area	29.0%	out of area
	10.8%	looking	16.7%	looking	24.4%	looking
	2.8%	walking	7%	flight	10.7%	eating
	1.9%	grooming	1.2	walking	2.0%	walking
	---		1.0%	hopping	---	
	---		1.0%	eating	---	

Each of the nine behaviors was recorded (named in descending order of appearance) in response to the four tested sounds: raven, hiss, stomp and whip played once.

**Table 2:** Time budget for experiment 2.

Signal	Minute 1 (pre-signal)		Minute 2 (post-signal)		Minute 3 (post-signal)	
5 s foot stomp	80.1%	eating	68.3%	looking	62.1%	out of area
(12x/min)	9.7%	looking	11.3%	eating	17.6%	looking
	3.8%	relaxing	5.8%	hopping	7.0%	hopping
	3.5%	walking	4.2%	out of area	4.5%	relaxing
	1.3%	grooming	3.0%	relaxing	4.5%	eating
			1.9%	alert	1.4%	walking
			1.2%	walking	0.6%	flight
			1.0%	flight		
5 s whip crack	48.5%	eating	71.2%	out of area	100%	out of area
(12x/min)	20.6%	looking	9.6%	flight		
	22.7%	relaxing	2.7%	hopping		
	3.5%	walking	1.6%	looking		
	4.5%	grooming	1.0%	alert		

Each of the nine behaviors were recorded (named in descending order of appearance) in response to the foot stomp or whip crack signal played at 12x/minute over minutes 2 and 3.

**Table 3:** Time budget for experiment 3.

Signal	Minute 1 (pre-signal)		Minute 2 (post-signal)		Minute 3 (post-signal)	
30 s whip crack	63.1%	eating	44%	looking	42.3%	looking
(2x/min)	21.7%	relaxing	33.7%	out of area	43.5%	out of area
	5.8%	looking	5.3%	flight	13.7%	eating
	5.1%	walking	4.4%	alert	0.3%	grooming
	2.3%	grooming	3.3%	eating	0.2%	walking
	---		0.8%	hopping	---	
15 s whip crack	77.2%	eating	69.3%	looking	40.1%	looking
(4x/min)	16.1%	looking	12.3%	eating	29.7%	eating
	4.3%	relaxing	5.1%	out of area	24.6%	out of area
	1.1%	walking	3.6%	hopping	2.7%	walking
	0.9%	grooming	2.6%	flight	1.5%	hopping
	0.5%	hopping	0.8%	walking	1.0%	grooming
	---		0.5%	alert	0.1%	flight
	---		0.1%	grooming	---	
3 s whip crack	68.5%	eating	46.4%	out of area	65.2%	out of area
(20x/min)	22.6%	looking	23.9%	looking	21.3%	looking
	3.3%	relaxing	8.5%	eating	12.3%	eating
	3.6%	walking	7.7%	flight	1.2%	walking
	2%	grooming	1.0%	hopping	---	
	---		0.7%	walking	---	

Each of the nine behaviors was recorded (named in descending order of appearance) in response to a whip crack signal played at three different rates over two minutes.

**Table 4:** Ethogram of kangaroo behaviors used for these experiments.

Behavior	Description of behavior	PC 1	PC 2	PC 3	PC 4	PC 5
Relax	Animal lies on the ground, head up with eyes open	0.03	−0.03	0.06	**−0.98**	0.02
Eating (**P1**)	Any active uptake of food (grazing and browsing)					
Chewing (**P1**)	Animal chews its food	0.07	−0.29	**−0.92**	0.13	−0.01
Regurgitate (**P1**)	Animal regurgitates food					
Crouched (**P1**)	Animal is standing in pentapedal position					
Grooming	Self grooming or interaction between animals grooming each other	0.01	−0.05	0.01	0.01	**−0.79**
Looking	Animal stands up on hind legs and tail and looks around, head and ears turning	−0.02	**0.92**	0.19	0.07	−0.00
Alert	Animal stands on hind legs and tail- more vertical body posture than looking, almost frozen state, usually only ears are moving.	**0.65**	−0.01	0.28	0.17	0.32
Walking	Pentapedal movement at a slow pace	0.07	0.21	−0.04	0.14	**−0.54**
Hopping	Bipedal movement, medium pace, animal hopping on hindlegs, body bend forward	**−0.69**	0.18	0.19	0.16	0.22
Flight 10 (**P2**)	Fast bipedal movement, body held vertical/upright. Up to 10 m distance before stop					
Flight 25 (**P2**)	As above, but up to 25 m distance before stop	0.21	−0.44	**0.60**	0.13	−0.03
Flight 50 (**P2**)	As above, but up to 50 m distance before stop					
Out of area	Animals moving more than 50 m away from the original position	**−0.57**	−0.46	**0.54**	0.10	0.11
Play (S)	Playful boxing					
Sleep (NR)	Animal lies on the ground, head down with eyes closed					
Touch (NR)	Any form of touching between individuals (non-aggressive)					
Aggressive (NR)	Any aggressive touch/movement or growl					
	**Eigenvalue**	1.81	1.42	1.20	1.07	1.01
	& Total variance explained	**20.17**	**15.75**	**13.38**	**11.88**	**11.23**

The weightings of the five Principal Components factors are indicated (values in bold show a weighting ±0.54).

P: pooled behaviors for analyses (P1: crouching, eating, chewing, and regurgitating were grouped into ‘eating’; P2: the three flight distances were grouped into ‘Flight’).

S: scarcely observed→ not analyzed.

NR: not recorded.

PC factor 1 (PC1; alert, hopping, moving) accounted for 20% of the total variance in the data and was explained by animals being *alert* (positively correlated with PC1) or *hopping* or moving *out of the area* (negatively correlated with PC1). For all three experiments, there was a significant time effect (Figure S1): there was little of this behavior shown in minute 1, but after auditory signal playback, animals showed an increase in *alert* behavior or *hopped* to move *out of the area* (particularly marked in experiment 2).

PC factor 2 (PC2; looking; 16% of the total variance) was positively correlated with animals *looking*. This factor showed variable responses across the three experiments, with significant signal x time interactions for all three experiments. In experiment 1, the foot stomp and raven call initiated the greatest degree of *looking* behavior, while the *hiss* generated a single side-ways or backwards hop followed by increased *looking*. The whip crack elicited the least amount of *looking* (and the greatest amount of locomotion – Figure S1d). In experiment 2, repeated playback of the f*oot stomp* resulted in increased *looking* from kangaroos; by contrast repeated playback of the *whip crack* did not result in increased looking because the animals had already made their decision and were taking *flight*, moving *out of the area* (Figure S1e). In experiment 3, the intermediate playback rate (4x/min) initiated more *looking*, but the faster playback (20x/min) resulted in less *looking* behavior (animals had already made their decision and were taking *flight*, moving *out of the area*; Figure S1f).

PC factor 3 (PC3; cease eating, flight, movement out of area; 13% of the total variance) was described by animals stopping *eating* (negative correlation with PC3) and taking *flight* and moving *out of the area* (both positively correlated with PC3). For all three experiments, there was a significant time effect (Figure S1g-i): animals stopped *eating* (minute 1) and instead took aversive action: taking *flight* and moving *out of the area*. In experiment 1 (Figure S1g), there were a significant signal and signal x time interaction effects – kangaroos showed little alteration to these behaviors after playback of the *hiss* (even returning to *eating*) or *raven call*. Changes in PC3 (cease eating, flight, movement out of area) were most marked for playback of the *whip crack*; this signal was the most effective in generating *flight* and movement *out of the area*. Increasing the rate of playback of the *whip crack* in experiment 2 (Figure S1h) resulted in greater aversive behavior, triggering a flight response during the first 10–20 s with 71% of the animals moving *out of the area* by minute 2, and 100% being *out of the area* in minute 3. This contrasts with playback of the *foot stomp* where only 4.2% of animals had left the area by minute 2, and 62.1% by minute 3. There was no signal x time interaction for experiment 3 (Figure S1i), since all treatments resulted in animals taking *flight* and moving *out of the area*; this was most marked for the highest rate of playback (significant signal effect).

PC4 and PC5 quantified ‘relaxed’ behavior that disappeared after exposure to the auditory signals. PC factor 4 (12% of the total variance) was negatively correlated with animals *relaxing*. There were significant time effects for PC4 (relaxed) across all three experiments, with animals pre-signal (minute 1) showing a range of *relaxing* behavior, but little or none of this behavior post-playback (this was particularly noticeable in experiment 3 (Figure Sl). PC factor 5 (relaxed;11% of the total variance) was negatively correlated with animals *grooming* or *walking*. As for PC4, we recorded significant time effects for PC5 across all three experiments, with animals pre-signal (minute 1) showing a range of *grooming* or *walking* behavior, but little or none of this behavior post-playback (there was no significant signal or signal x time interaction effects).

### Do the number of animals, or identify of a mob, influence our analyses?

Alert and eating behaviors, the only behaviors that had sufficient variability to warrant statistical analysis, were analyzed as an indication of whether the action of a few animals could influence the mob, or whether the identify of a mob, or number of individuals could impact our results. Neither *alert* nor *eating* behavior was affected by group size (number of individuals in the mob; table 5). The effect of trial number on *eating* behavior showed a trend, however (*P* = 0.055; table 1), with animals returning to *eating* earlier if they had already been subjected to multiple trials previously. There was also a trend for effect of group number (*P* = 0.059) upon *alert* behavior, while group number significantly affected *eating* behavior (table 5); each individual's response was linked to how the others in their group responded. Trial number was an important factor in terms of a measurement of habituation. There was no effect of trial number upon the degree of *alert* behavior demonstrated (table 5). Thus, there was no indication that animals altered their degree of alertness in response to being tested over multiple days.

**Table 5:** Statistical results for the behaviors alert and eating.

	alert		eating	
	*F*	*p*	*F*	*p*
Stimulus (fixed independent variable)	**6.60**	**0.009**	2.50	0.112
Trial no. (covariate)	1.61	0.226	4.41	0.055
Group size (covariate)	0.16	0.700	<0.01	0.936
Group no. (random factor)	1.73	0.059	**2.13**	**0.015**

Data were analyzed in a mixed model ANOVA. Values in bold indicate effects where p<0.05.

### Experiment 1: Do kangaroos change behavior patterns in response to natural and artificial auditory signals?

We recorded significant differences between signals in experiment 1, with different responses due to the different signals (Figure S1, table 1). For example, the *foot stomp* initiated more *looking*, the *hiss* elicited a instant *hop* followed by looking, whilst the greatest aversive reaction (stopping *eating*, *flight* and movement *out of the area*) were recorded in response to the *whip crack* (one of our artificial signals) followed by the *foot stomp* (a bio-acoustic signal).

### Experiment 2: Are kangaroos more likely to habituate to artificial signals than natural signals?

Kangaroos did not show any sign of habituation to either playback of the *whip crack* or *foot stomp* when signals were played frequently (12x/min). Although they first spent time *looking* in response to the *foot stomp* (Figure S1e), both signals elicited an aversive reaction, with animals stopping *eating*, taking *flight* and movement *out of the area* (Figure S1h).

### Experiment 3: Are kangaroos sensitive to the rate of stimulus presentation?

The only significant signal x time interaction for experiment 3 was for PC2 (Figure S1f): in response to the intermediate rate of playback, kangaroos spent more time *looking*, while they did not do so for the faster frequency of playback.

## Discussion

Contrary to previous reports [17], [32] and to our predictions, kangaroos responded most aversively to the playback of an artificial signal, the bull *whip crack*, and failed to rapidly habituate to this novel sound. Repeated playback of this signal (experiment 2) stopped animals *eating* and elicited a greater number of *flights*, with animals moving *out of the area*. *Flight out of the area* in response to repeated stimulus has similarly been recorded for other studies [28], [31], although changing the rate of stimulus playback (experiment 3) did not show a significant effect of signal playback rate (non-significant signal x time interaction effect for PC3 (eating cessation, flight, movement out of area) for this experiment).

Playbacks of natural foot stomps were not entirely ineffective, just less dramatic than expected [17]. In a natural environment, animals may require visual feedback to elicit the strongest repellent response from audible alarm behaviors. Notably, in experiment 2, the foot stomps elicited an increase in *looking* behavior, which was less evident in response to playback of the whip crack by comparison. Interestingly, the biological control sound (raven call) also increased *looking*; this signal may therefore have been correctly perceived as a bird call since the animals looked upward and toward the tree line rather than toward the speaker. This natural response provided confidence in the quality of our recordings and playback apparatus. Likewise, the artificial hiss may have also been perceived as a biologically meaningful snake sound. During the first 5 s following playback, most animals *hopped* to the side and backwards upon hearing the noise from front facing speakers. Interestingly, no animals took *flight* or *left the area* following this sound. This acute response is similar to how semi-wild kangaroos respond to snakes (Carol Lander, Roo Gully Wildlife Sanctuary, pers. comm.); after kangaroos hop away a short distance, the snake would no longer be perceived as threatening.

The disparate types of responses to each sound (sideways *hop*, *look* up, or *flight*) provide some support for our hypothesis that the animals were at least partly reacting to biologically-relevant information rather than amplitude alone. Some animal sounds, such as alarm calls containing biologically relevant information, are characterized by rapid rise times and high amplitudes [33]. Thus, it will be challenging to determine whether the rise times- or the biologically relevant information – elicited the observed reactions.

We were surprised that kangaroos did not rapidly habituate to either the *whip crack* or the *foot stomp*, and that an increase in playback rate did not influence habituation during the time of our trials. The lack of habituation to highly evocative stimuli is consistent with some studies [21], [29], but in contrast to others [28], [31]. Surprisingly, the most rapid playback was not the most evocative; more animals took flight and left the vicinity when the signal was played 4 x per minute (though not significantly so), than either the slower or faster rates. This implies that, among some behaviors, deterrent efficacy does not always increase with a decrease in the inter-stimulus period. This is important because typically a reduction in inter-stimulus period could result in more opportunities for habituation [28], this is no longer a concern when the lower frequency of playback elicits better responses. Further research will be necessary to address this interesting question.

### Conclusion

While deterrents based on single modalities may appear promising, we need to investigate ways to prolong time to habituation. One way to avoid habituation may be by combining different signal modes – i.e. developing multi-modal deterrents. For instance, wolves (*Canis lupus*), American black bears (*Ursus americanus*) and coyotes (*Canis latrans*) rapidly habituate to exposure to visual deterrents; however, when used in combination with acoustic deterrents, visual deterrents are more effective [34]. Similarly, strobe lights used in combination with sirens protect pastured sheep from coyote predation for at least three months [35]. The mix of modalities may work because it is more difficult for animals to habituate to a complex and fearful multi-modal stimulus. Given that multi-modal stimuli may be more effective, we propose that a combination of both natural and artificial signals could be investigated, since we have demonstrated efficacy of both natural and artificial acoustic signals in the present study.

Despite the poor performance of artificial ultrasonic deterrents on influencing the behavior of kangaroos in previous studies, we were able to use an artificial auditory stimulus to generate persistent aversive behaviors. This is important because non-invasive methods are needed to assist the management of kangaroos where they encroach on human habitats. Even acute applications could reduce the need for culling or mitigate the numbers of animal-vehicle collisions. The natural foot stomp was no more aversive, nor less likely to result in habituation, than the bull whip crack. Thus, both novel and bio-acoustic cues may be used concurrently to mask predictability and therefore delay habituation. Increased frequency of delivery did not result in significantly more flights from the area, however, and managers should understand that ‘more is not always better’ when it comes to application of animal deterrents. Deploying sensory-based deterrents requires more knowledge of the process of habituation and how to delay its onset. Ultimately, a combination of sensory cues (novel and natural, or perhaps auditory and olfactory modalities) may result in the better long-term performance for non-invasive animal deterrents.

## Materials and Methods

### Study Site and Animals

Experiments were conducted between October 2007 and April 2009 at Roo Gully Wildlife Sanctuary (RGWS), Boyup Brook, Western Australia, a semi-natural setting, 270 km SE of Perth (33° 49′18.41 S, 116° 22′ 52.34 E). Forty-eight semi-wild western grey kangaroos had free range of a 9.7 Ha fenced area. Kangaroos ranged in age from 12 months to 12 years, with most individuals between the ages of 3 and 5 years. All individuals were recognizable by differences in facial, tail, toe and/or pelage. Animals had *ad libitum* access to water, herbage and shrubs and supplemental pellets and grains were freely available at four feeding stations. All experiments were in compliance with the National Health and Medical Research Council (NHMRC) of Australia's code of practice for protecting animal welfare during research; ethics approval was granted from Curtin University (AEC 02-08).

### Technical equipment for sound recording and playback

This experiment was designed to be salient to kangaroo hearing. Bender [17] showed the hearing of *M. giganteus* to be most sensitive between 1.7–3.5 kHz. Sounds were captured with a Sennheiser MKH816 shotgun microphone (40 Hz to 20 kHz) and recorded onto a Marantz PMD671 compact flash recorder (sampling rate 24 bit, 96 kHz). In addition, to maximize foot stomp recordings, a Raven PZM-30D microphone (range 20 Hz to 20 kHz) with a 10 m long extension cable was placed at ground level to increase the angle of sound capture and to increase the chance of recording seismic sounds. Sounds were edited with Raven Lite 1.0 (Ithaca, NY) and AVS Audio Editor (Boston, Massachusetts). Sounds were broadcast through a large active subwoofer (Genelec 7060B; 20 Hz to 120 Hz with an output of 113 dB Sound Pressure Level, SPL), and a smaller full-range speaker (Genelec monitor) was set to overlap with this range (120 Hz to 20 kHz; output of 106 dB SPL) producing an accurate frequency response of 20 Hz to 20 kHz. Playback intensity was measured by a digital sound level meter (Q 1264, IEC 651 Type II, 30–130 dB SPL). Speaker amplitude was adjusted depending on the individual signal and distance of the speaker to the kangaroos, so that volumes were selected to match their natural amplitudes (these varied by stimulus, see below).

### Acoustic signal selection and recording

After pilot experiments, four sounds were selected: two artificial and two natural (biologically-relevant) signals. Sound recordings of artificial sounds were obtained between October 2007 and December 2007. Natural sounds were recorded between January 2008 and September 2008.

All recordings were made during ideal weather conditions (dry, no wind gusts over 2 m/s). Prior to trials, we used the sound level meter at varying distances from the speakers to determine the volume setting to ensure that subjects heard each stimulus at the appropriate amplitude, irrespective of their distance from the speakers. Broadcast amplitude varied by stimulus and distance to subjects. The ‘best’ signal was selected for each exemplar based on ‘clarity’ by examining the audiograms and selecting the one with the sharpest contrasts.

1. The *hiss* is an artificial sound produced by a 1 s burst from an aerosol spray can. The gas release from various 100 g spray cans was recorded and the one considered to sound the most ‘snake-like’ to human ears was selected. Subjects in the center of the mob heard hisses at 50 dB SP; a ‘natural’ amplitude that mimicked snake hisses.
2. The natural amplitude of a bull *whip crack* (an artificial sound created by cracking a bull whip) required a slightly higher volume. Subjects heard the bull *whip crack* at 70 dB SPL in the center of the mob. Several recordings of the bull whip were made and the recording that generated the greatest response (sharpest crack) during the pilot was selected for further trials.
3. Australian raven caws were selected as presumed non-threatening control sounds. Multiple recordings of *raven calls* were made with the clearest recording selected for trials. Subjects in the center of the mob heard the *raven call* at 50 dB SPL.
4. Western grey kangaroo *foot stomps* were recorded in natural settings when animals became startled. Among nine recordings of the foot stomp, the one that received the strongest response during the pilot was selected for trials. Subjects heard the foot stomp at 60 dB SPL reflecting their natural signal strength.

### Experimental trials

Experimental trails were conducted between December 2008 and April 2009. Trials were carried out between 06:00–09:00 h and 18:00–20:00 h local time because this corresponds with the time that this population of kangaroos most actively forage (Carol Lander, personal communication). Ambient conditions and wind are known to affect feeding patterns and vigilance behavior [36], thus trials were only run when weather conditions were similar (temperatures ranged between 14–23°C and wind speeds, measured by an anemometer, between non-detectable and 4.5 m/s). Other factors affect vigilance such as group size and structure [37] and local predatory pressure [38], these variables were controlled as far as possible in our experiment through control of predator presence by fox baiting and restriction of access by dogs, and by including mob size as a covariate when analyzing data with sufficient variability to do so (alert and eating).

Experimental trials were preceded by six weeks of desensitizing subjects to the speakers and to the observers. The trolley, which held the playback equipment, was placed 7–30 m from subjects with both speakers facing the focal subjects. No sounds were played over this time. The desensitization period was considered successful when the observers and equipment could approach within 7–30 m of the focal animal without causing it to leave the area. During trials, the volume was then adjusted depending on distance to the mob. We waited at least 15 min after approach and setup for subjects to resume normal behavior (i.e. mostly foraging and resting behaviors without any signs of agitation). Filming (Sony mini DV Digital Handycam Camera, 120x digital zoom, Carl Zeiss Vario-Sonnar, super steady shot, DCR-TRV22E) commenced with the purposes of identifying as many individuals in the group as possible (i.e. zooming in on face and different body parts like ears, tail and feet). When the animal identification filming was complete and the minimum resting period had elapsed, filming as a group shot was carried out for at least a minute. This first minute represents the pre-treatment behavior of the kangaroos. After the first minute, the treatment (acoustic signal) was triggered by remote control and the group was filmed for another two minutes to record changes in behavior. We conducted three experiments to study salience and habituation.

#### Experiment 1: Do kangaroos change behavior patterns in response to natural and artificial auditory signals?

A random choice of each of the four signals was played back once, at the end of the first minute.

#### Experiment 2: Are kangaroos more likely to habituate to artificial signals than natural signals?

We broadcast the *whip crack* or *foot stomp* signals repeatedly at regular intervals (12x/min) for the entire minute 2 and minute 3 periods.

#### Experiment 3: Are kangaroos sensitive to the rate of stimulus presentation?

We broadcast the *whip crack* repeatedly at either 2x/min, 4x/min or 20x/min for the entire minute 2 and minute 3 periods.

### Data Analysis

At least 22 individuals participated in each experiment (experiment 1: n = 24; experiment 2: n = 22; experiment 3: n = 23). The response of each subject to each of the different signals was quantified using JWatcher 1.0 [39]. Before the videos were scored, maps were constructed displaying the exact position the individual kangaroos within the field of view to ensure the identity of each focal individual.

We were not able to carry out statistical analysis on all behaviors independently because there was a lack of variation in some responses (for example, by definition there were no animals scored as ‘*out of area*’ in minute 1, nor were there any animals taking *flight* at this time – the trials would only commence if all animals were settled after the approach of the observers and equipment). We therefore extracted factors using principal component (PC) analyses calculated for the proportion of time individuals allocated to each behavior for each minute of each experiment. Five PC factors (Eigenvalues >1) were retained for analysis by repeated-measures ANOVA followed by Tukey's HSD post hoc tests. The independent factor was signal (treatment), and data for each of the 3 minutes were the repeated measure (time).

To analyze whether mob size, trial number or mob number had a significant influence on behavior for experiment 1, we fitted mixed-model ANOVAs for the two most distinctly different behavioral patterns: time spent *alert* and time spent *eating*. Mob size was the covariate, mob number was the random variable, and signal treatment was the independent variable. The dependent variable was the differential between minute 1 (pre signal) and minute 2 (post signal) time allocations.

## Supporting Information

Figure S1 Summary of five Principal Components (PC) factor scores derived from ethograms of nine kangaroo behaviors in response to playback of different auditory signals. In experiment 1 (a. left panel), single playback of four auditory signals was trialed (raven call, hiss, foot stomp and whip crack). In experiment 2 (b. central panel), repeated playback of the foot stomp and whip crack signals was tested (12x/min for minutes 2 and 3). In experiment 3 (c. right panel), varying rate of playback of whip crack was tested (treatments were broadcast at 2x/min, 4x/min and 20x/min for 2 min). Because the PC factor scores were calculated for all trials together, the scales of each PC factor are relative across the three experimental treatments. Values are mean ± 1SD.
(0.23 MB PDF)
