# Supplementary figures and images for: Sounds Scary? Lack of Habituation following the Presentation of Novel Sounds
Source: PLoS One. 2011 Jan 18;6(1):e14549. doi: 10.1371/journal.pone.0014549 (PMC3022648; doi:10.1371/journal.pone.0014549)

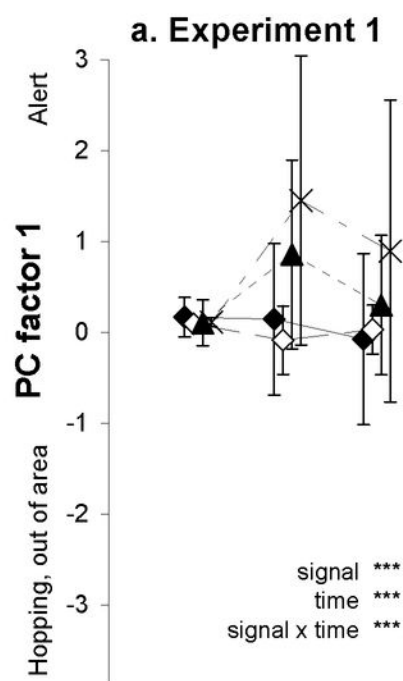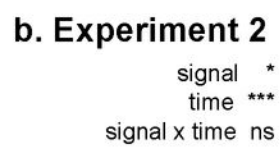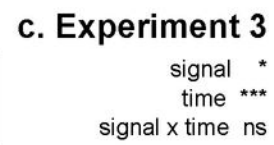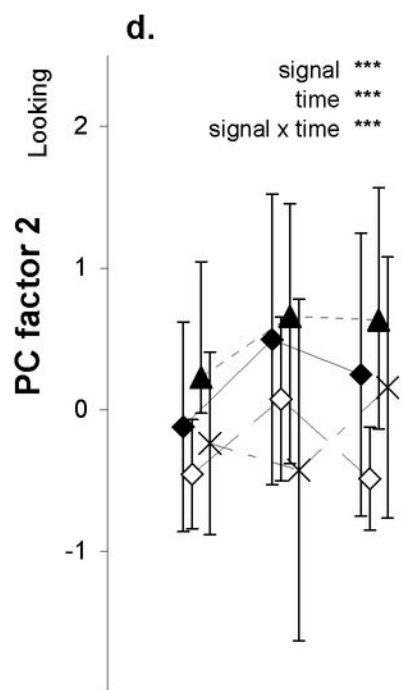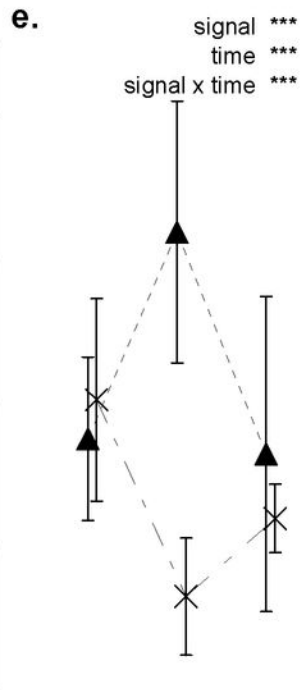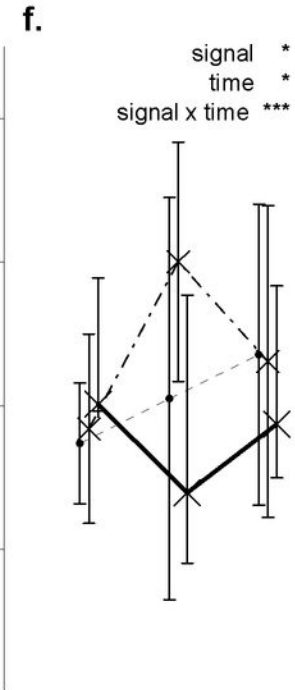

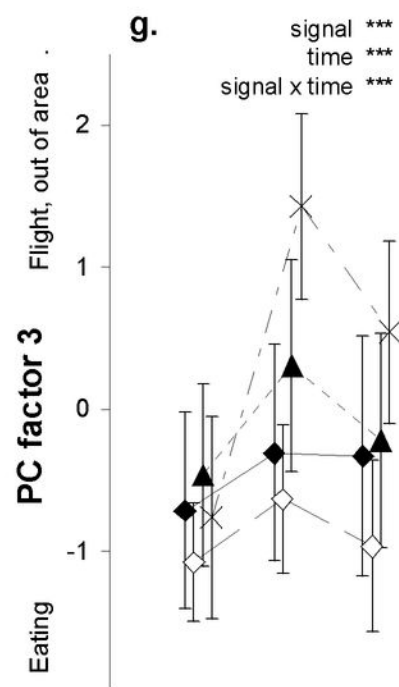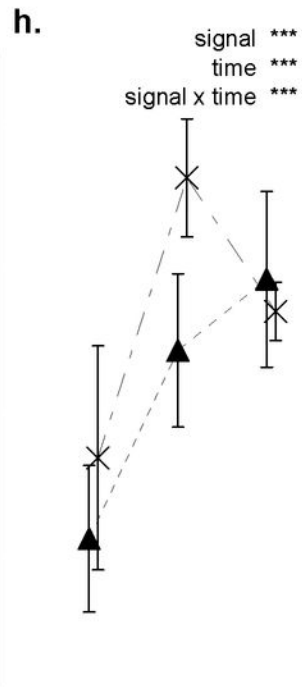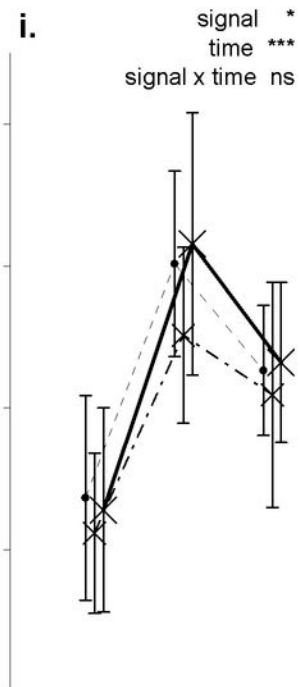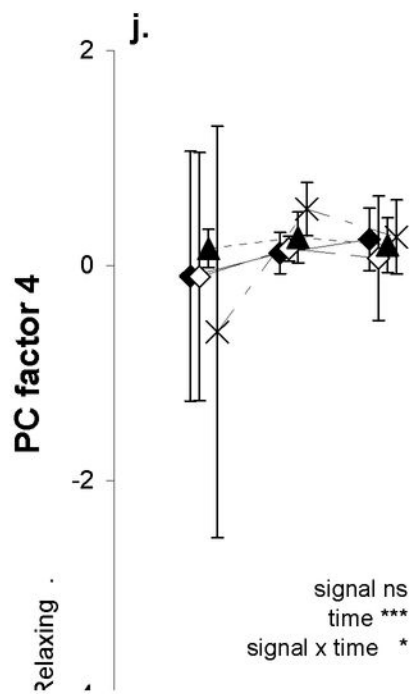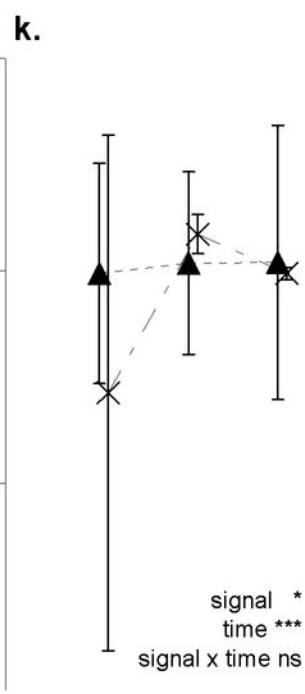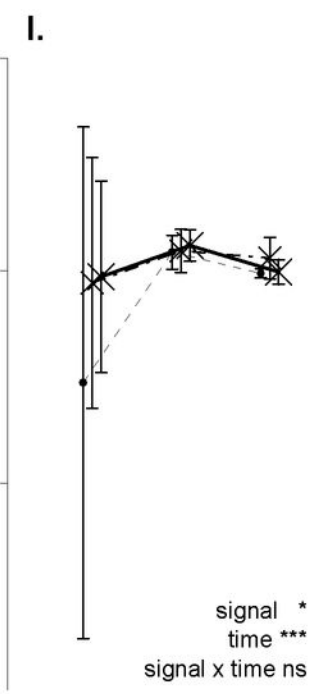

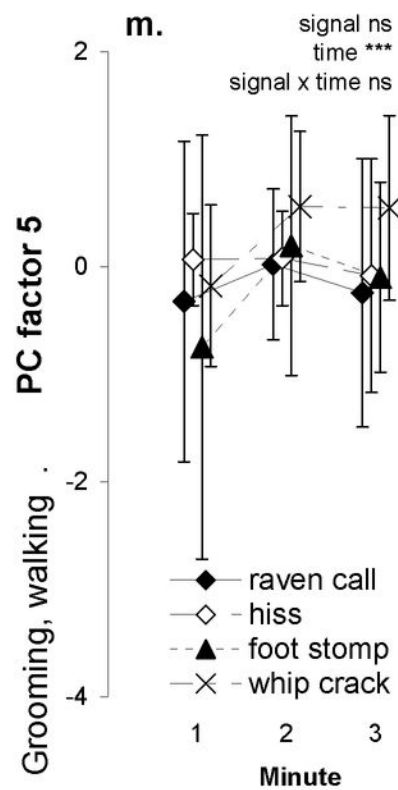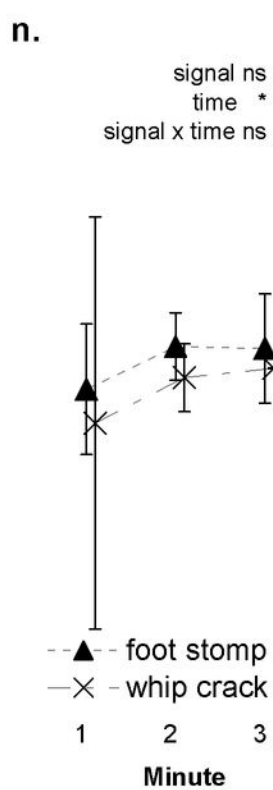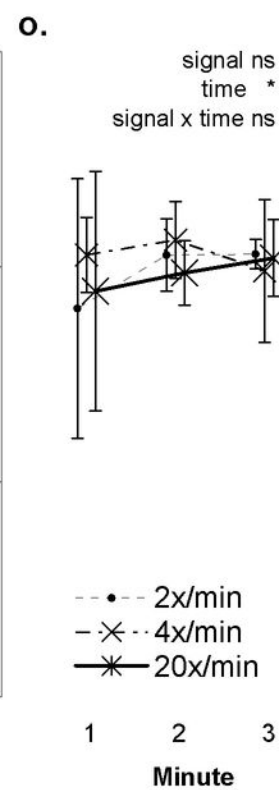

Supplement: Figure S1 — Summary of five Principal Components (PC) factor scores derived from ethograms of nine kangaroo behaviors in response to playback of different auditory signals. In experiment 1 (a. left panel), single playback of four auditory signals was trialed (raven call, hiss, foot stomp and whip crack). In experiment 2 (b. central panel), repeated playback of the foot stomp and whip crack signals was tested (12x/min for minutes 2 and 3). In experiment 3 (c. right panel), varying rate of playback of whip crack was tested (treatments were broadcast at 2x/min, 4x/min and 20x/min for 2 min). Because the PC factor scores were calculated for all trials together, the scales of each PC factor are relative across the three experimental treatments. Values are mean ± 1SD. (0.23 MB PDF) [file pone.0014549.s001.pdf]
